# Supplementary material for: Using Electric Stimulation of the Spinal Muscles and Electromyography during Motor Tasks for Evaluation of the Role in Development and Progression of Adolescent Idiopathic Scoliosis
Source: J Clin Med. 2024 Mar 19;13(6):1758. doi: 10.3390/jcm13061758 (PMC10971464; doi:10.3390/jcm13061758)

The Supplementary Material

Stimulation

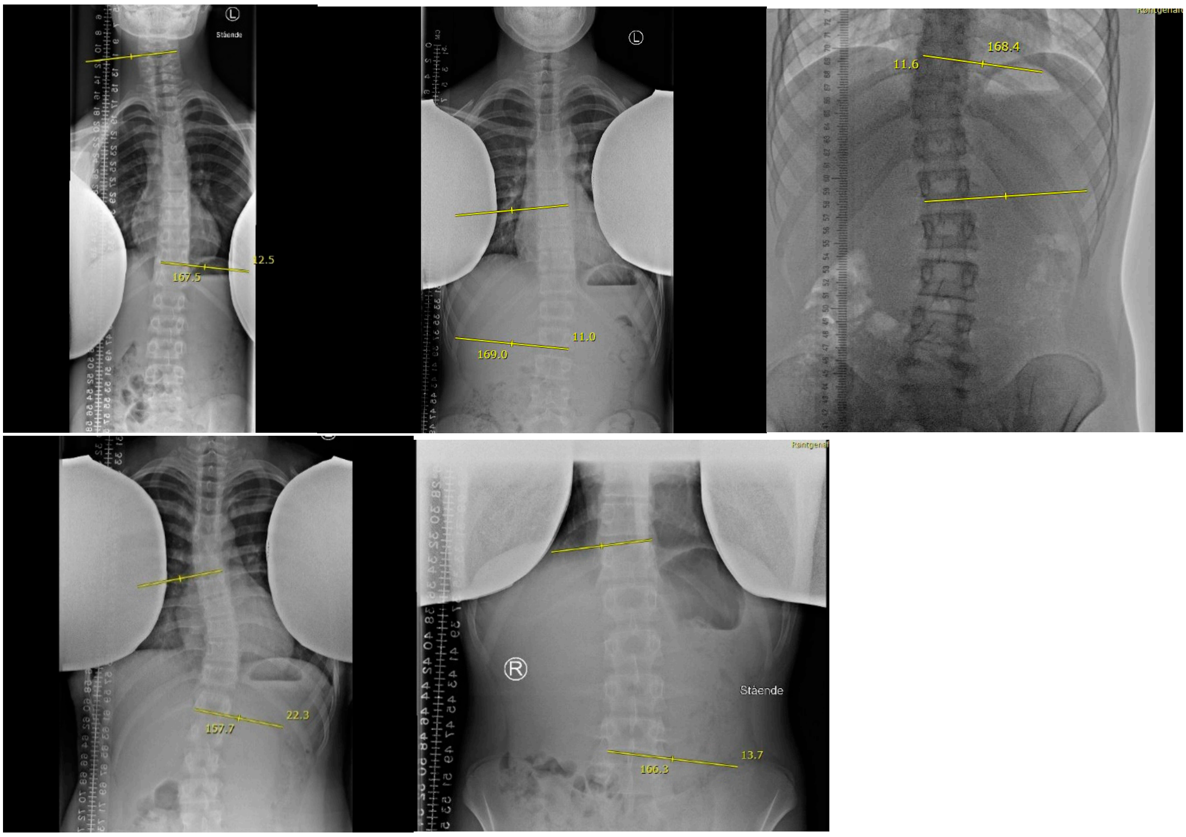

Figure S1. The initial AIS curves for 5 subjects for stimulation.

Subject 1 without (left) and with electric stimulation (right)

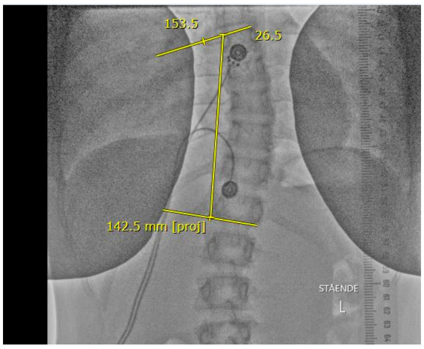

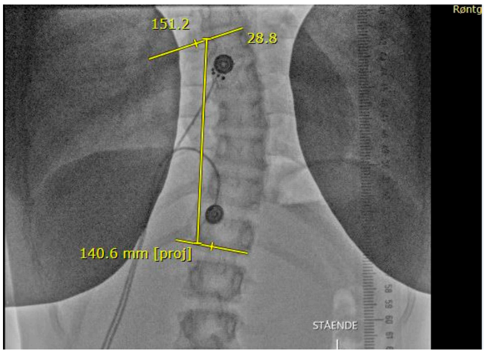

Subject 2 without (left) and with electric stimulation (right):

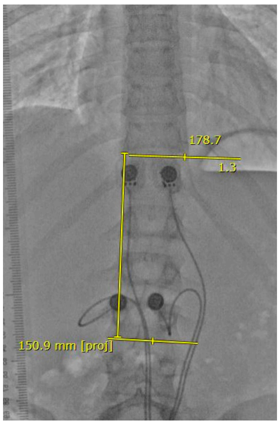

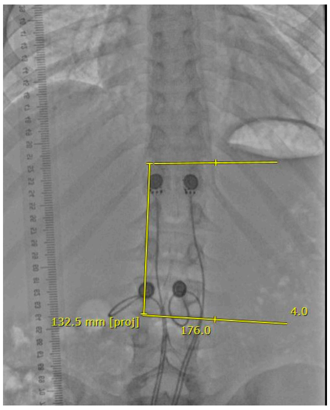

Subject 3 without (left) and with electric stimulation (right)

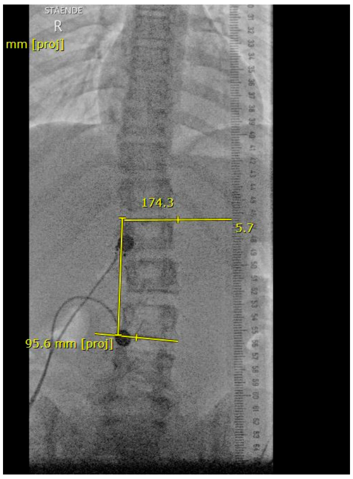

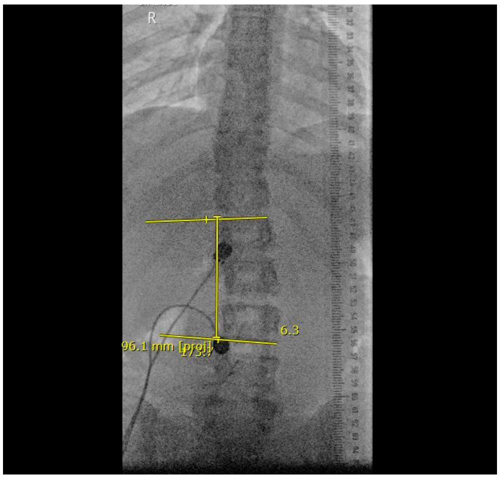

Figure S2. The AIS curves with and without stimulation for 5 subjects.

Subject 4 without (left) and with electric stimulation (right)

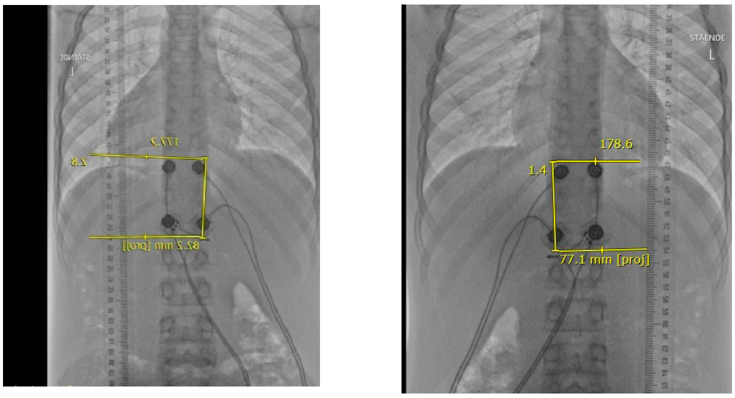

Subject 5 Lumbar without (left) and with electric stimulation (right)

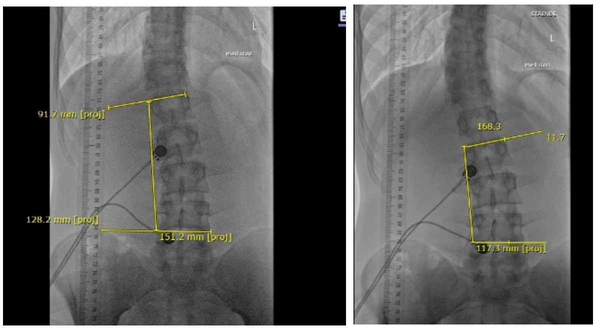

Subject 5 Thoracic without (left) and with electric stimulation (right)

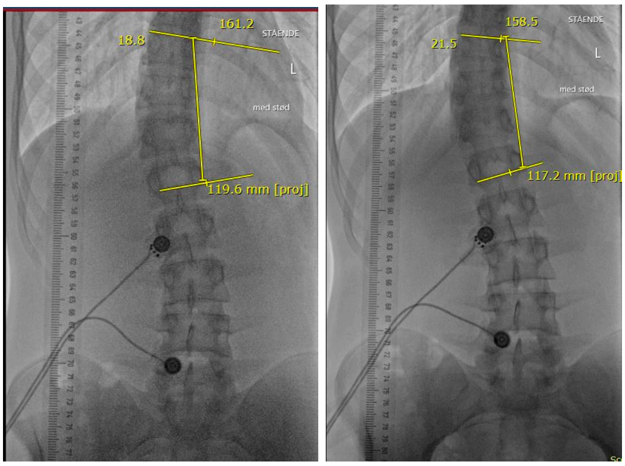

Cinematic recordings.

Val 4

9A74A3B6.mp4

Alb 2

BBEE134.mp4

Sar 1

496AC162.MP4

Hja 5

F08247C0.mp4

Din 3

Video S1. The initial and maximal stimulated AIS radiographs (Top) and the cinematic sequences for stimulation of the 5 subjects (Bottom)

EMG

E1. Description of the exercises and program of the instructions for the subjects:

The following exercise procedures were performed:

1. Lateral bending without arm swing to the right and stay (5 sec)

2. Lateral bending without arm swing to the left and stay (5 sec)

3. Lateral bending without arm swing to the right and return to standing (5 times)

4. Lateral bending without arm swing to the left and return to standing (5 times)

5. Lateral bending 2kg in each hand to the right and stay (5 sec)

6. Lateral bending 2kg in each hand to left and stay (5 sec)

7. Rotation and return to standing right (5 times)

8. Rotation and return to standing left (5 times)

E2. One example of EMG data for subject Sar for the performed exercises.

These EMG data were synchronized with the videos with repetitions of the exercises by manual evaluation as described.

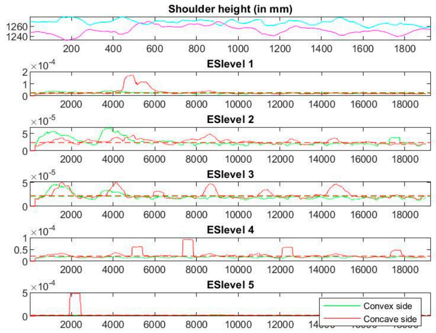

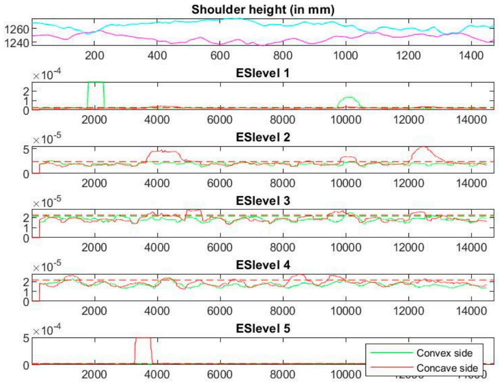

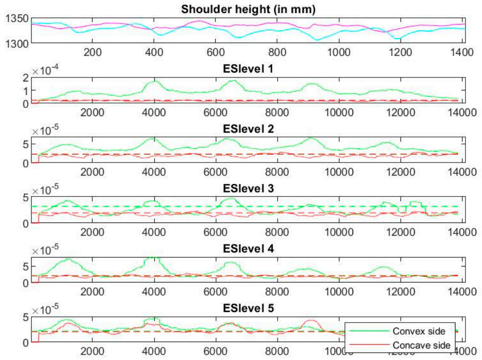

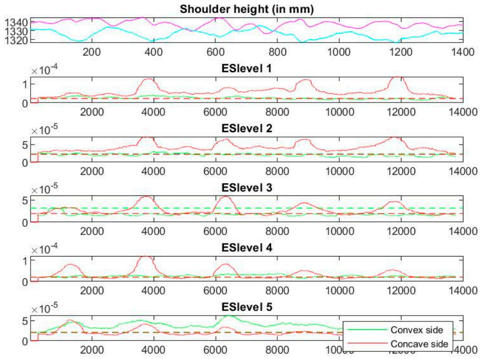

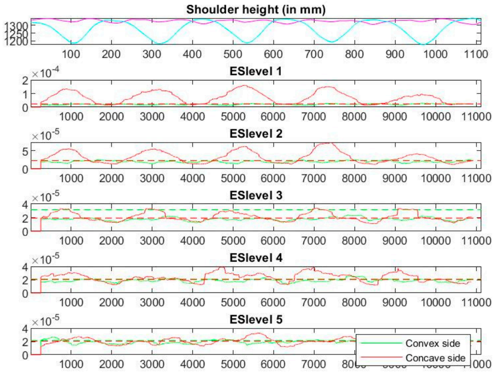

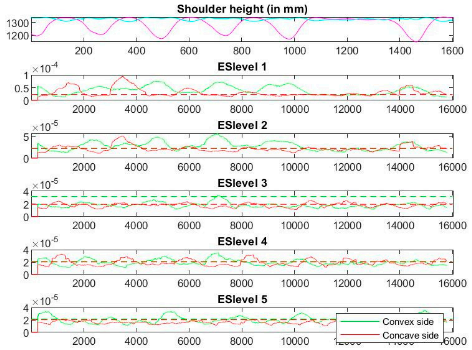

E3. Various ratios of the EMG

A. PEAK(ii) EMG convex / EMG convex+ EMG concave for lateral bending for left and right and rotation for left and right
B. PEAK (i) EMG convex / EMG concave for lateral bending for left and right and rotation for left and right
C. MEAN (i) EMG convex / EMG concave for lateral bending for left and right and rotation for left and right
D. MEAN (ii) EMG convex / EMG convex+ EMG concave for lateral bending for left and right and rotation for left and right

Upper: upper part of the primary curve/Mid: at the apex of the primary curve/Low: lower part of the primary curve

A. PEAK

Left rot

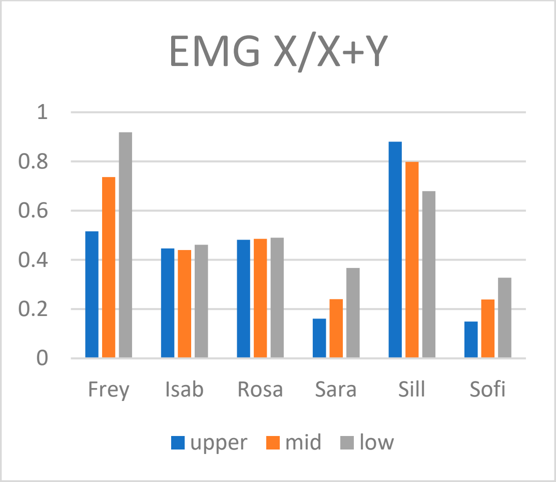

Right rot

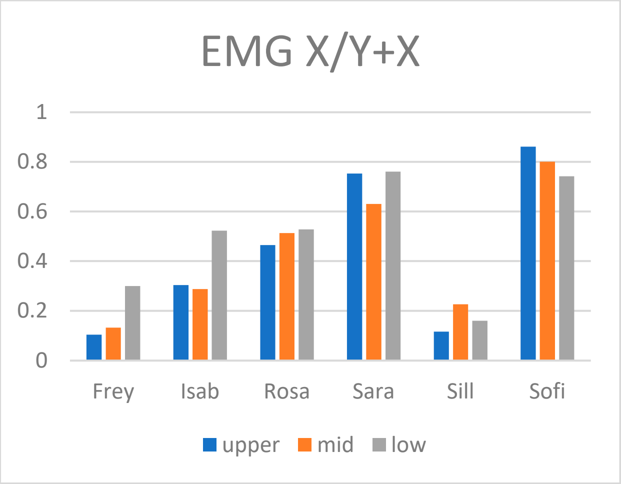

Left Lat flex

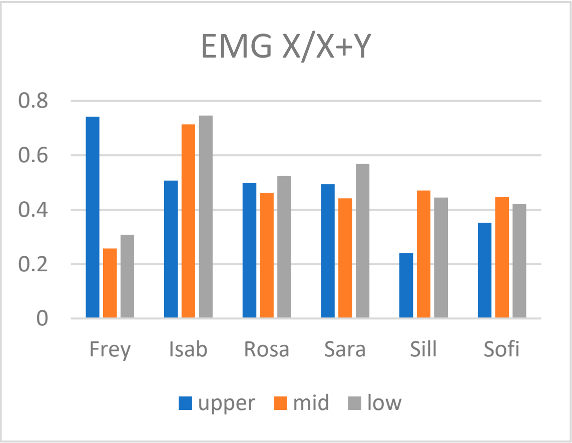

Right lat Flex

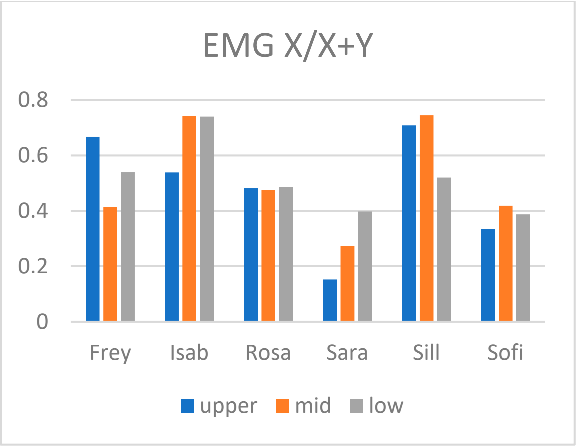

B. PEAK

Left rot

Right rot

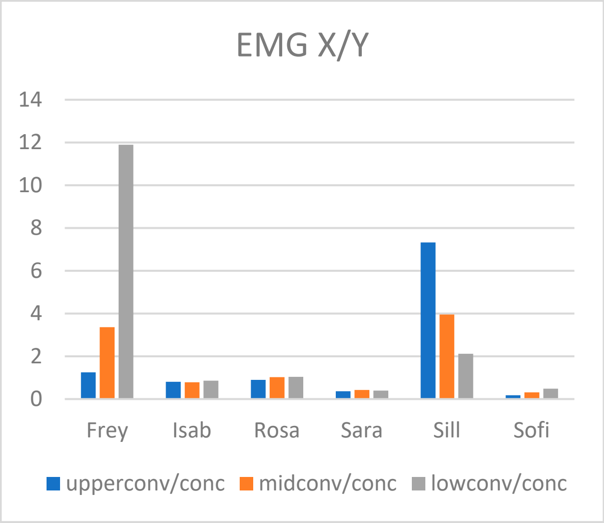

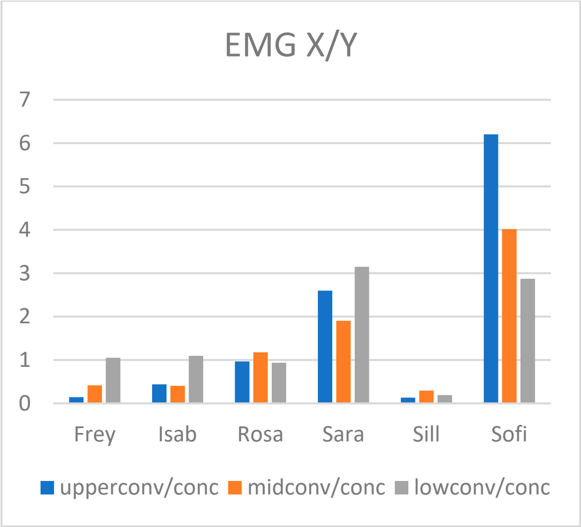

Left Lat flex

Right lat Flex

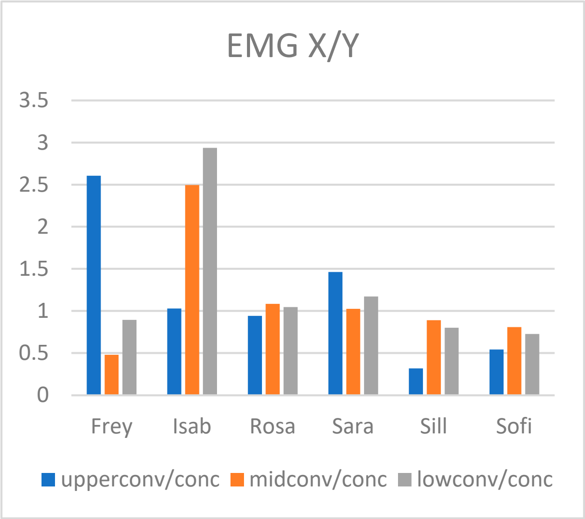

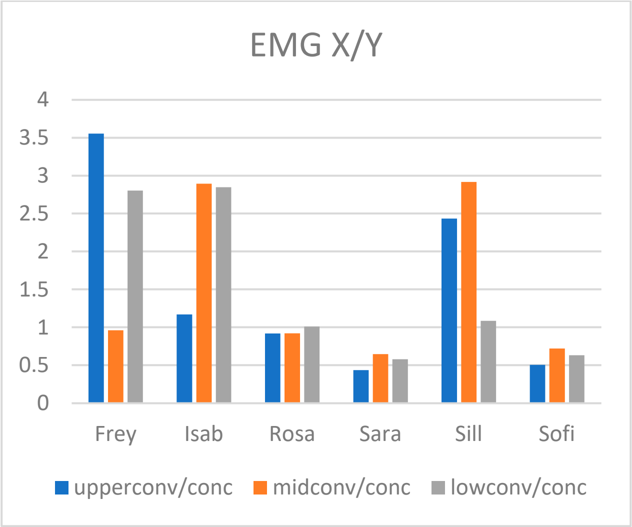

C. MEAN

Left rot

Right rot

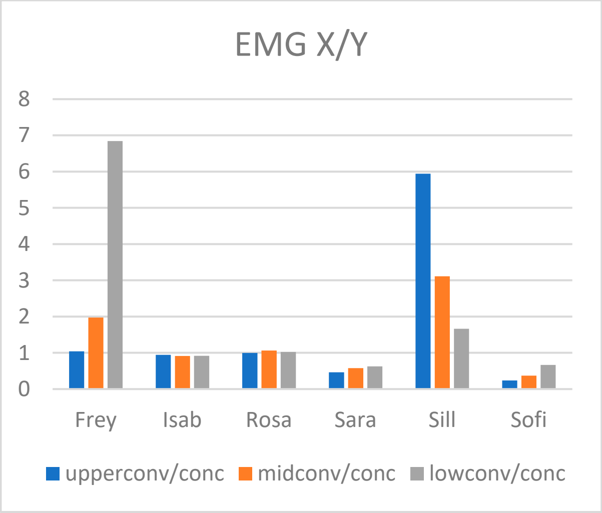

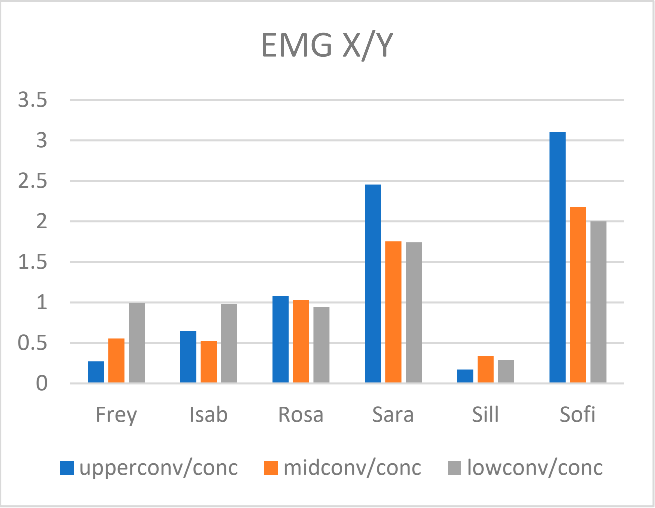

Left Lat flex

Right lat Flex

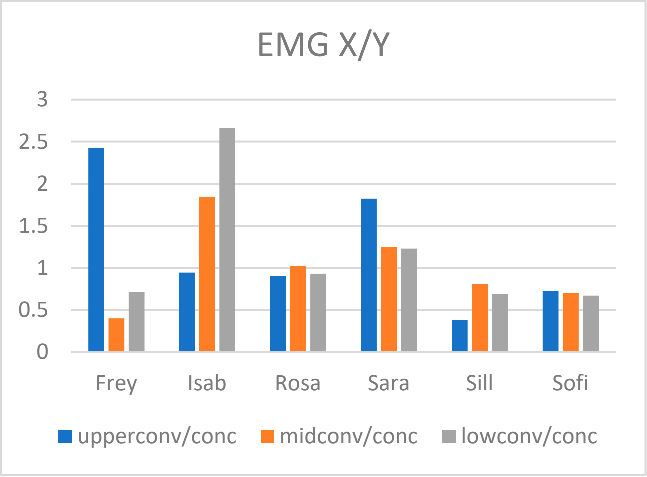

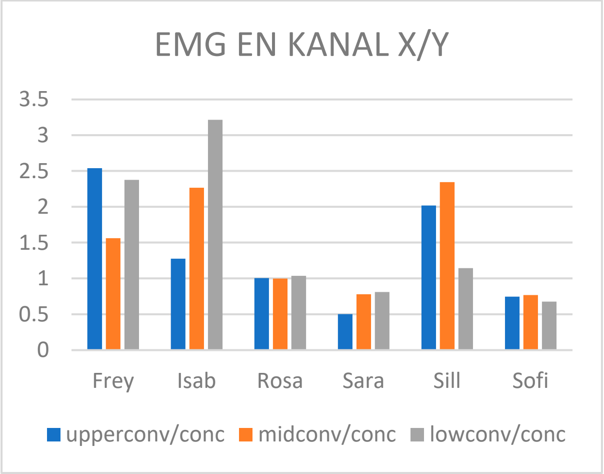

D. MEAN

Left rot

Right rot

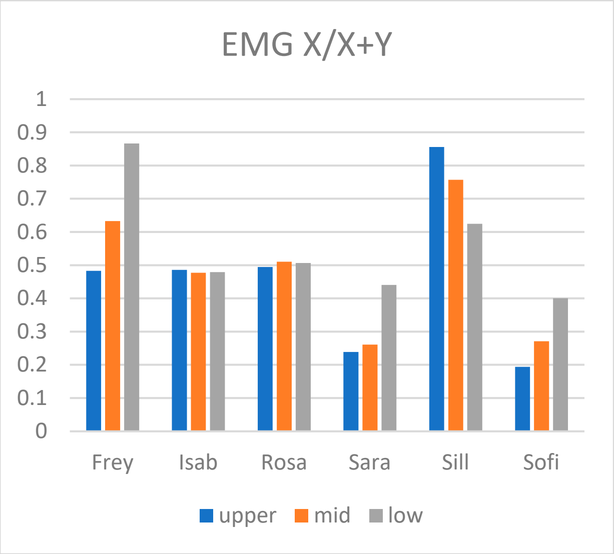

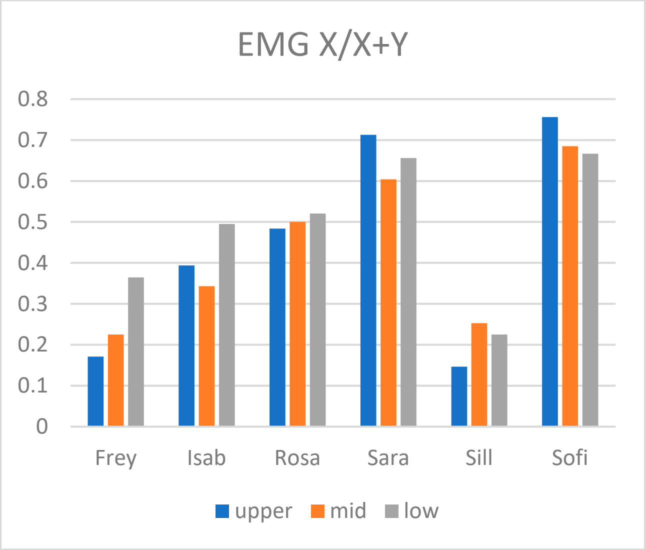

Left Lat flex

Right lat Flex

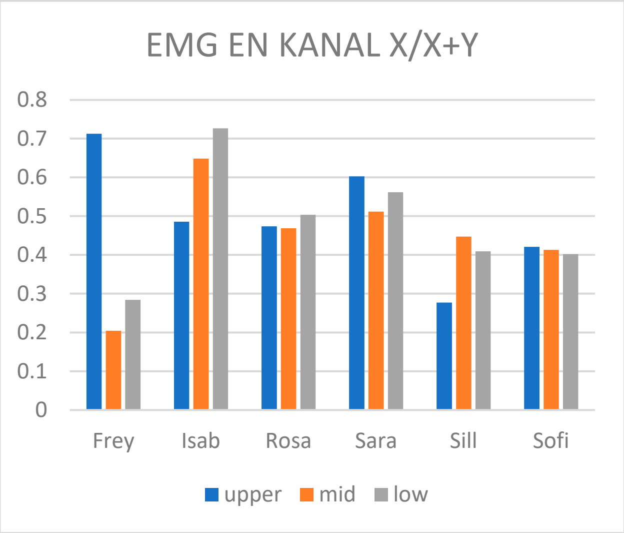

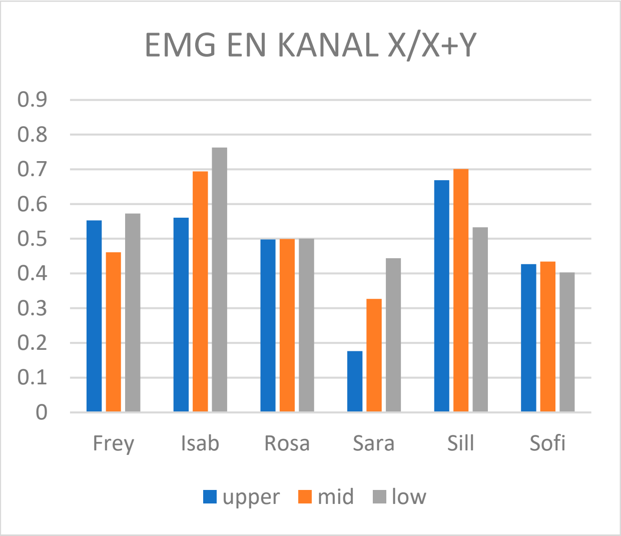

E. PEAK (i) EMG convex / EMG concave for lateral bending for left and right and rotation for left and right – when evaluated relative to the average ratio for the person.

F. PEAK(ii) EMG convex / EMG convex+ EMG concave for lateral bending for left and right and rotation for left and right – when evaluated relative to the average ratio for the person.

G. MEAN (i) EMG convex / EMG concave for lateral bending for left and right and rotation for left and right – when evaluated relative to the average ratio for the person.

H. MEAN (ii) EMG convex / EMG convex+ EMG concave for lateral bending for left and right and rotation for left and right – when evaluated relative to the average ratio for the person.

E. PEAK

Left rot

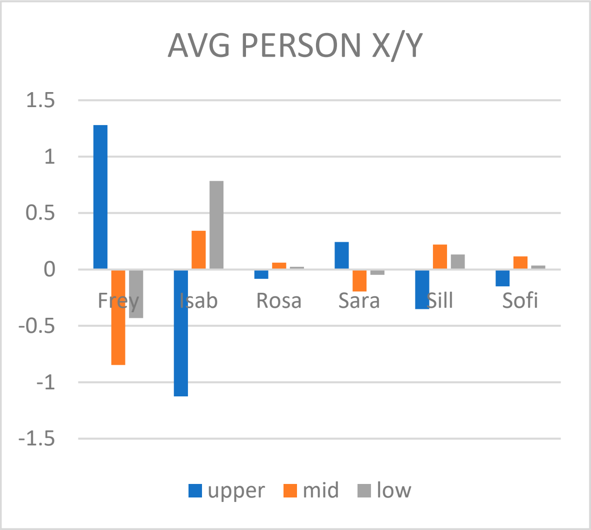

Right rot

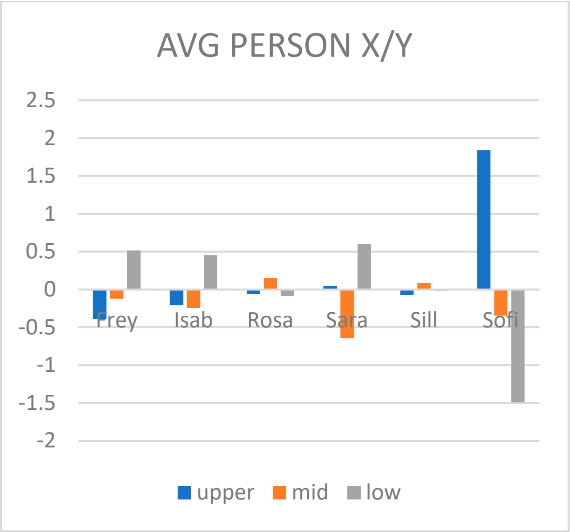

Left Lat flex

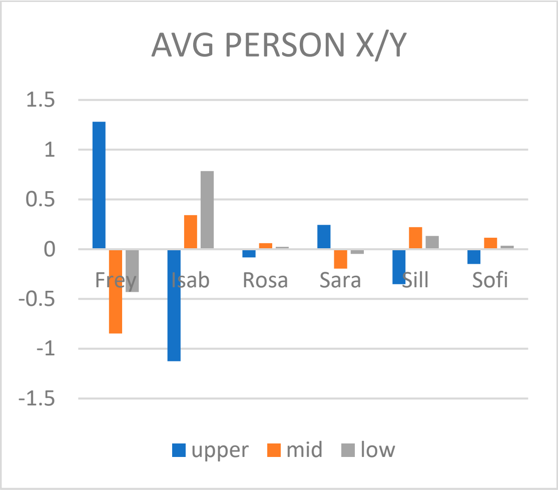

Right lat Flex

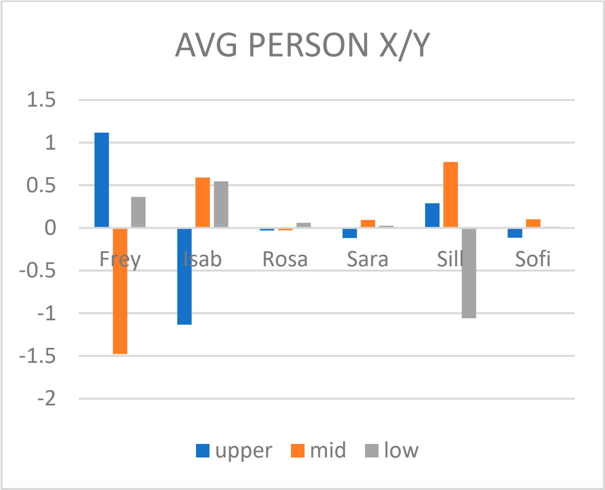

F. PEAK

Left rot

Right rot

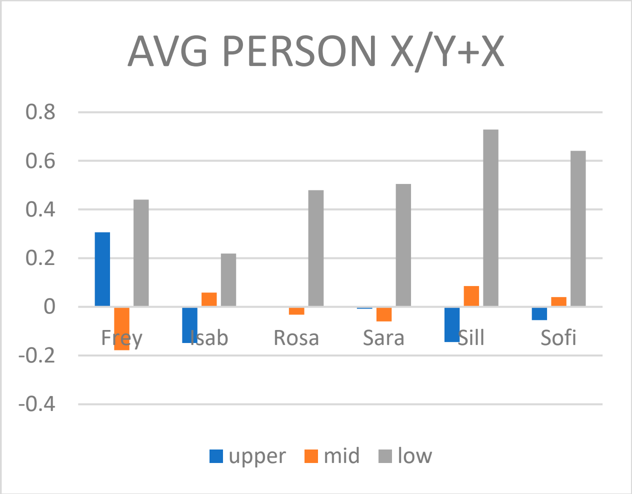

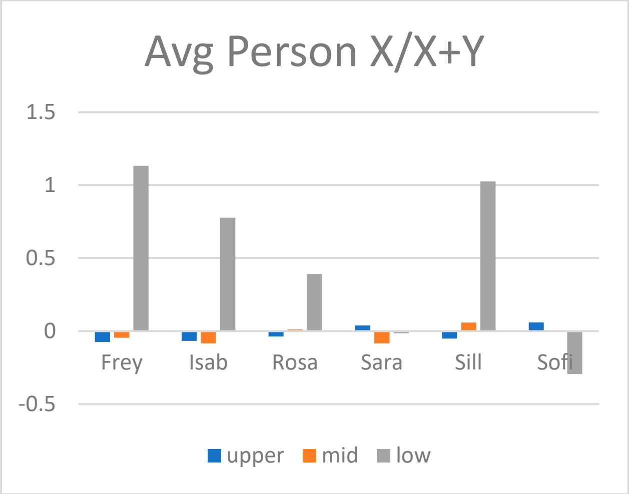

Left Lat flex

Right lat Flex

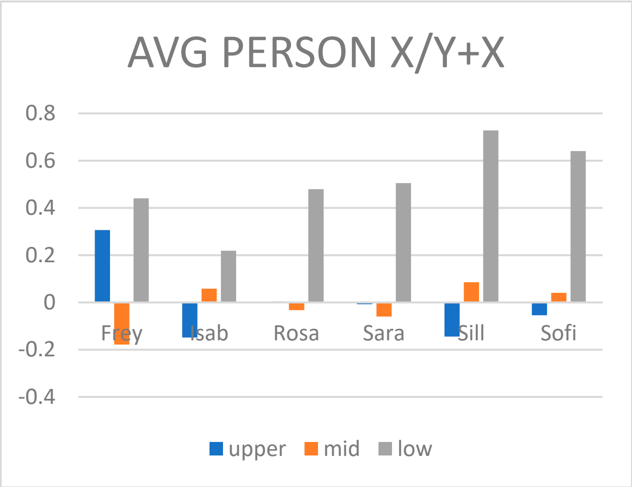

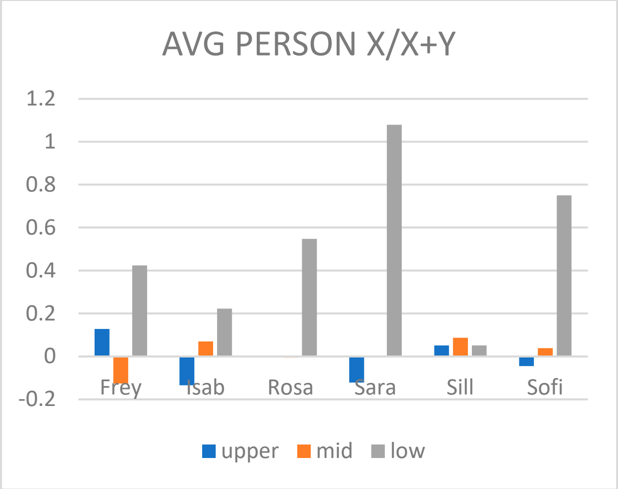

G. MEAN

Left rot

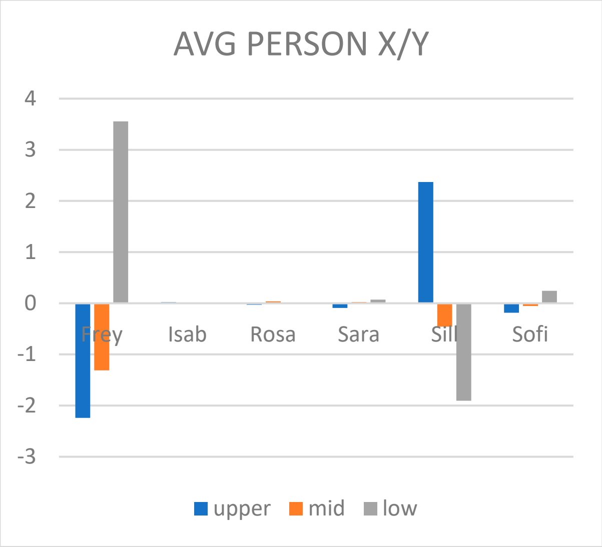

Right rot

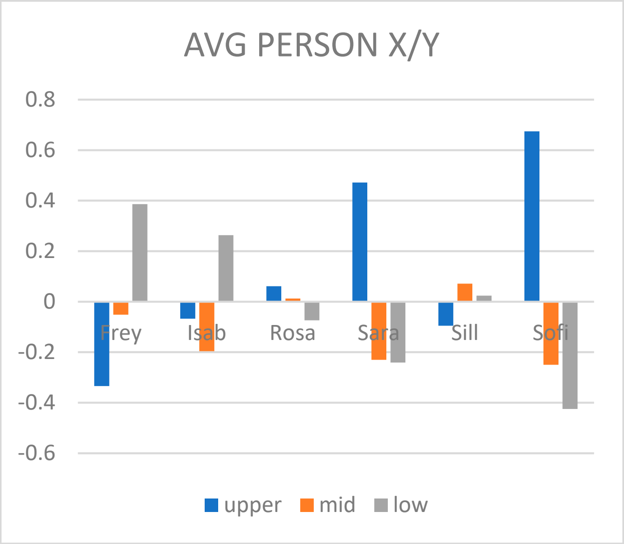

Left Lat flex

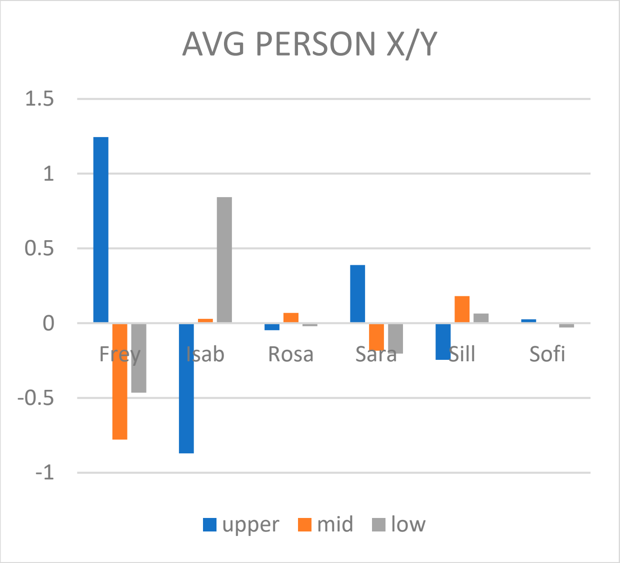

Right lat Flex

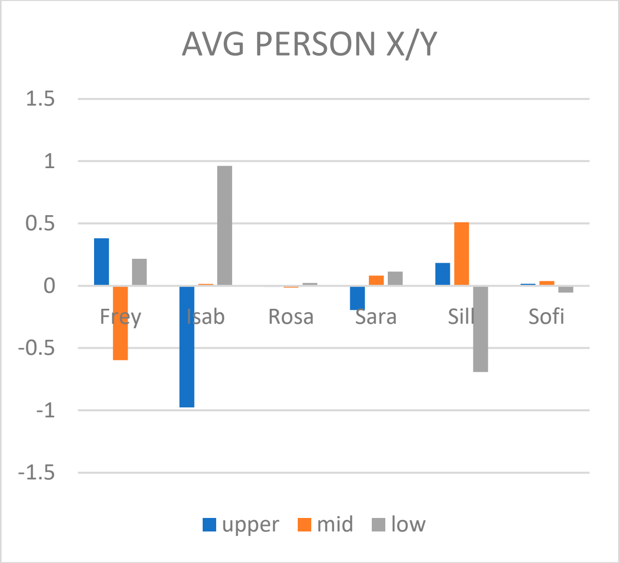

H. MEAN

Left rot

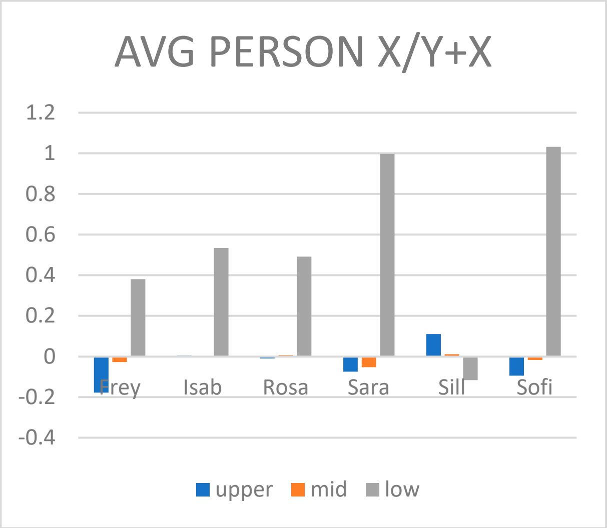

Right rot

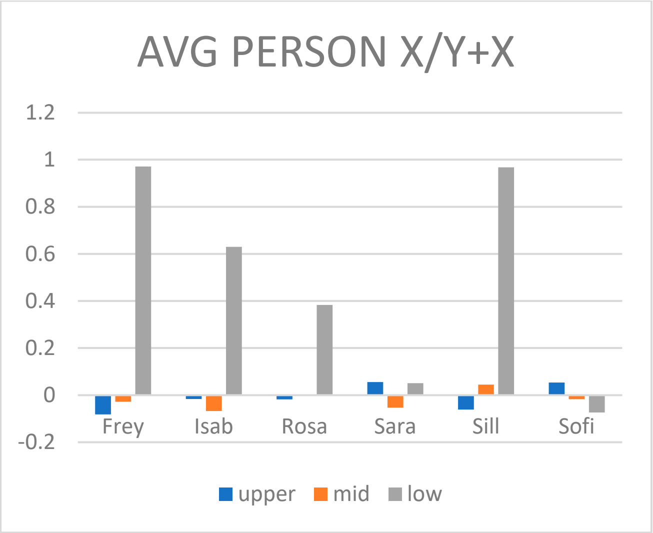

Left Lat flex

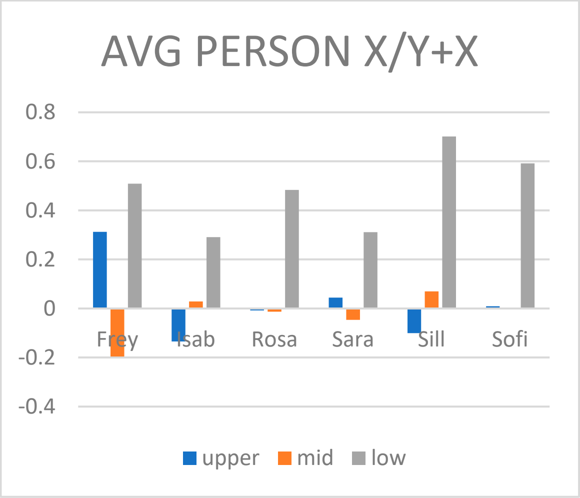

Right lat Flex

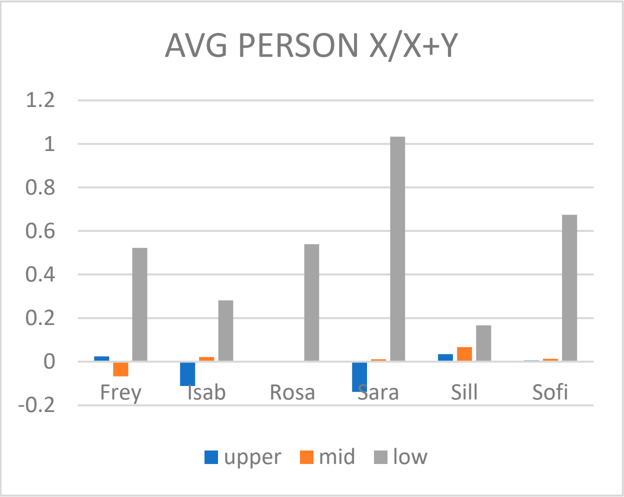

I. SUMMED TOTAL MEAN (i) EMG convex / EMG concave for lateral bending for left and right and rotation for left and right – when evaluated relative to the average ratio for the person.

J. SUMMED TOTAL MEAN (ii) EMG convex / EMG convex+ EMG concave for lateral bending for left and right and rotation for left and right – when evaluated relative to the average ratio for the person.

K. SUMMED TOTAL PEAK (i) EMG convex / EMG concave for lateral bending for left and right and rotation for left and right – when evaluated relative to the average ratio for the person.

L. SUMMED TOTAL PEAK(ii) EMG convex / EMG convex+ EMG concave for lateral bending for left and right and rotation for left and right – when evaluated relative to the average ratio for the person.

M. SUMMED TOTAL MEAN (i) EMG convex / EMG concave for lateral bending for left and right and rotation for left and right – when evaluated relative to the average ratio for the person FOR ABSOLUTE VALUES.

N. SUMMED TOTAL MEAN (ii) EMG convex / EMG convex+ EMG concave for lateral bending for left and right and rotation for left and right – when evaluated relative to the average ratio for the person FOR ABSOLUTE VALUES.

O. SUMMED TOTAL PEAK (i) EMG convex / EMG concave for lateral bending for left and right and rotation for left and right – when evaluated relative to the average ratio for the person FOR ABSOLUTE VALUES.

P. SUMMED TOTAL PEAK(ii) EMG convex / EMG convex+ EMG concave for lateral bending for left and right and rotation for left and right – when evaluated relative to the average ratio for the person FOR ABSOLUTE VALUES.

I. MEAN X/Y

Left rot

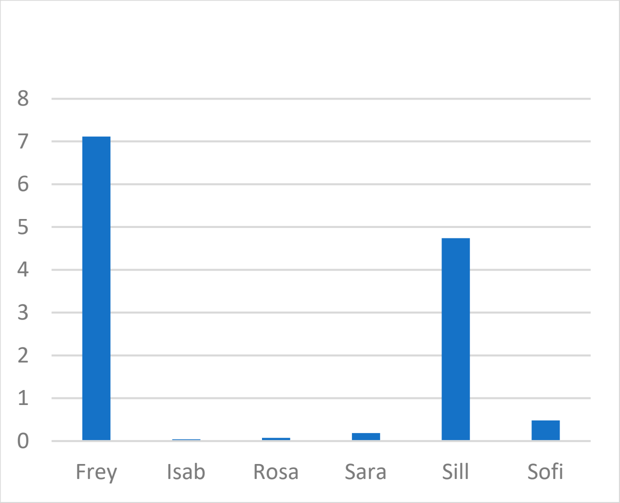

Right rot

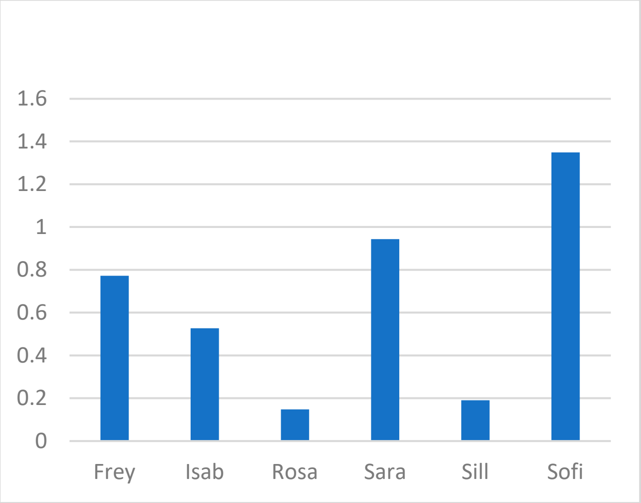

Left Lat flex

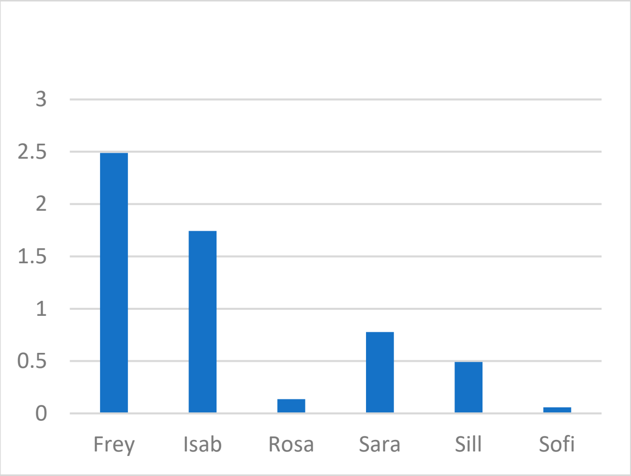

Right lat Flex

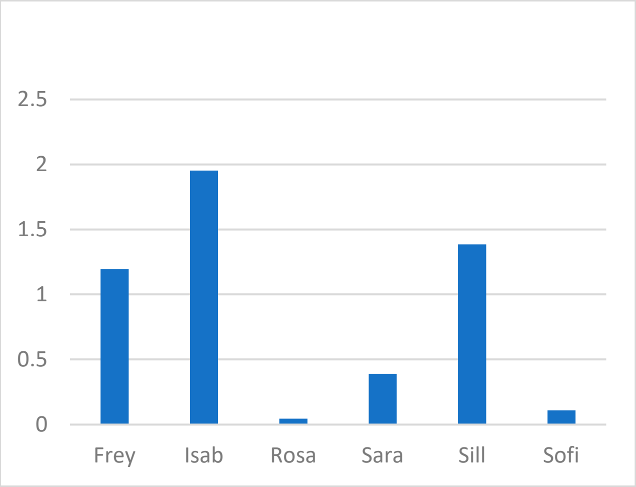

J. MEAN X/X+Y

Left rot

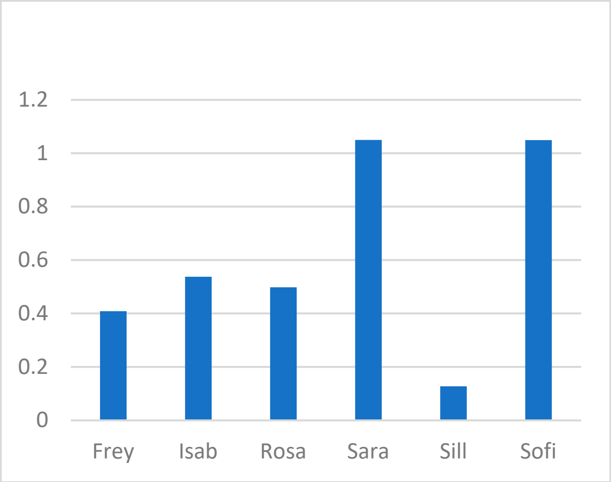

Right rot

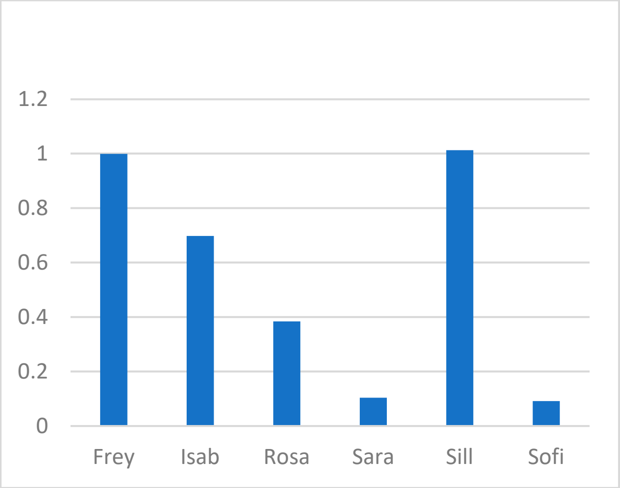

Left Lat flex

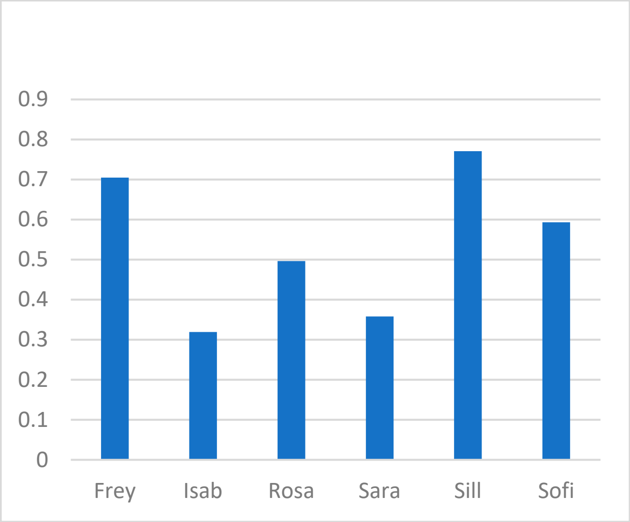

Right lat Flex

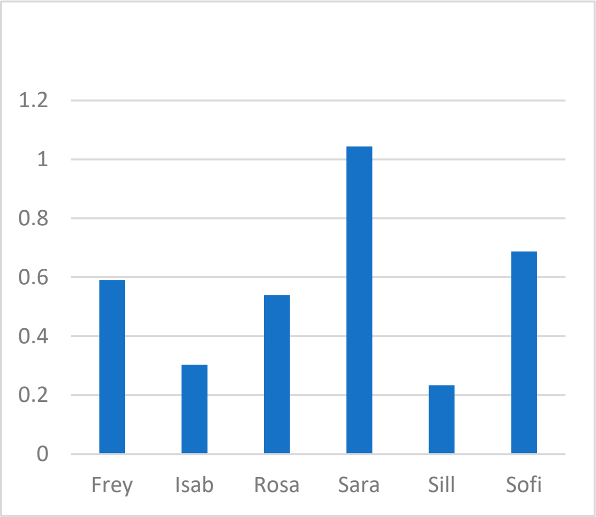

K . PEAK X/Y

Left rot

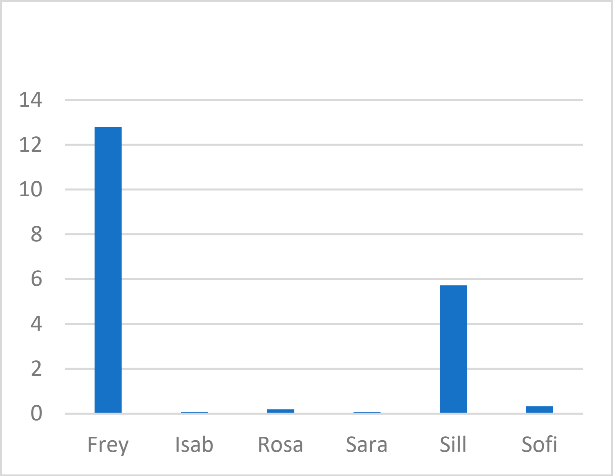

Right rot

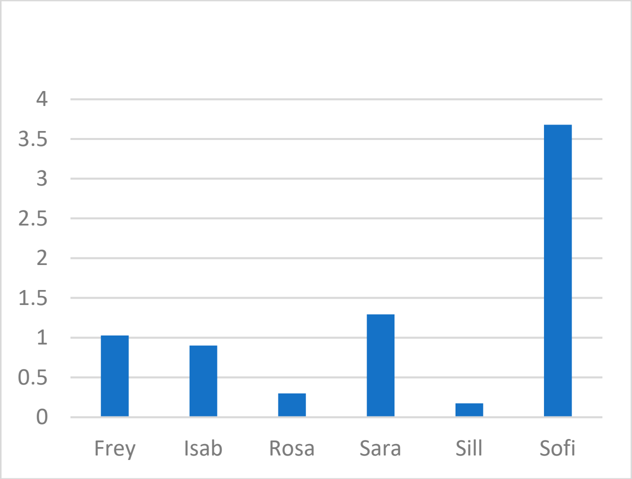

Left Lat flex

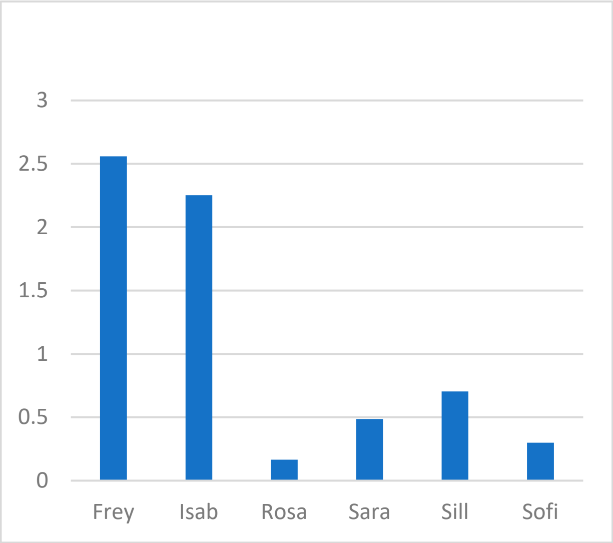

Right lat Flex

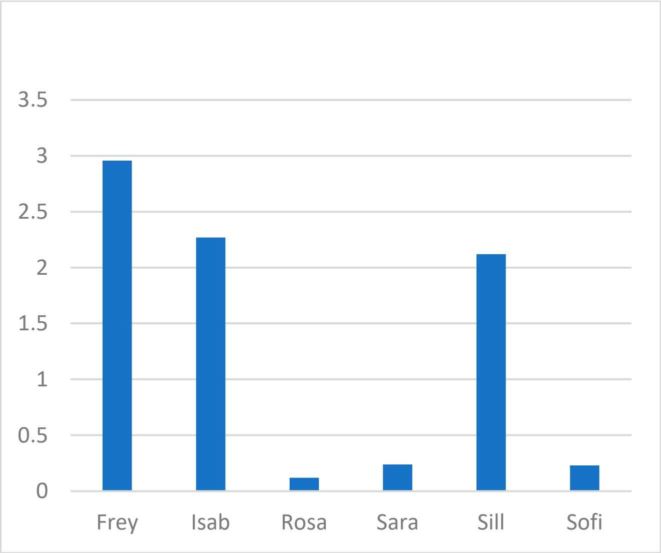

L.PEAK X/X+Y

Left rot

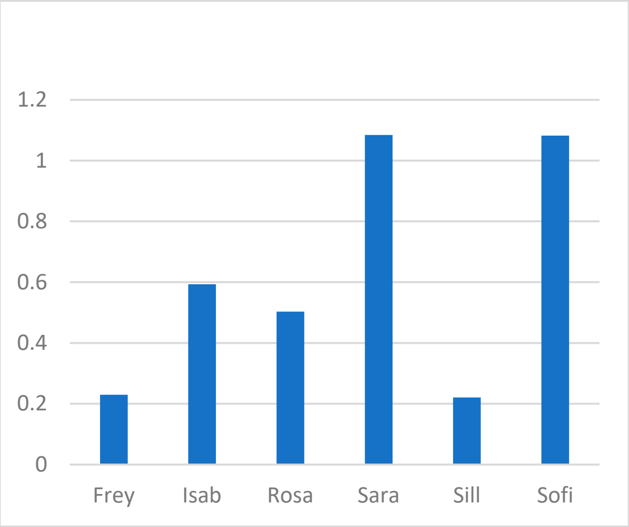

Right rot

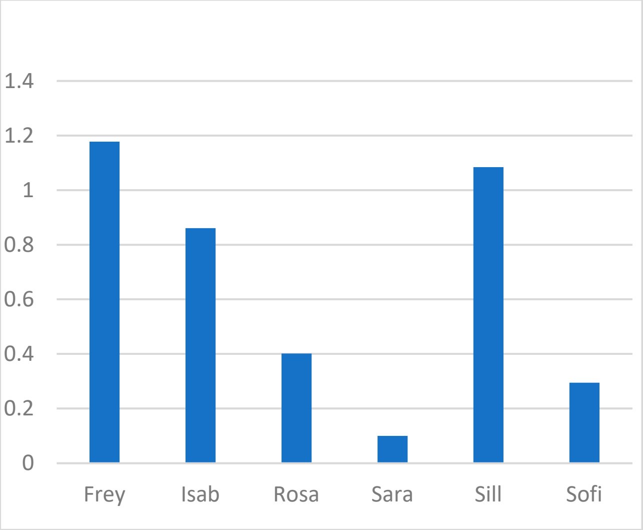

Left Lat flex

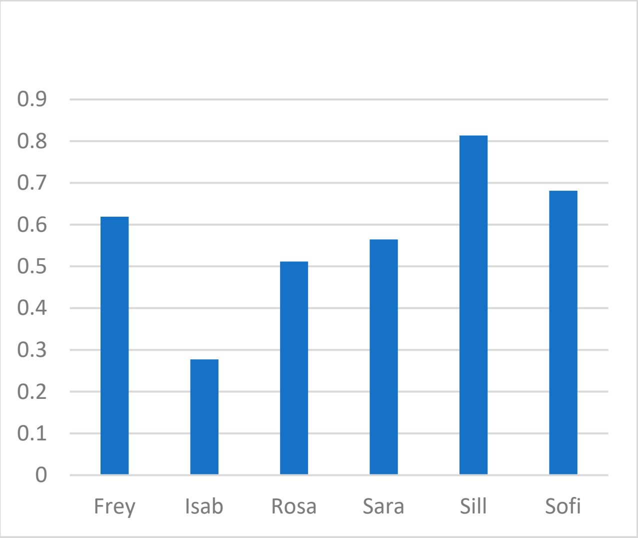

Right lat Flex

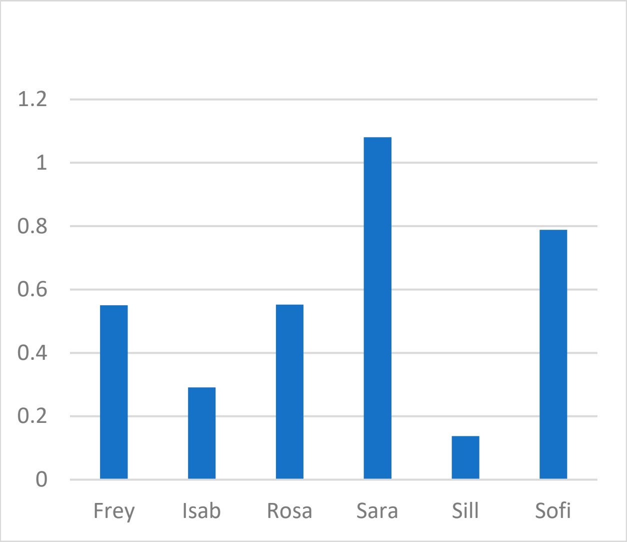

M. MEAN ABS

Left rot

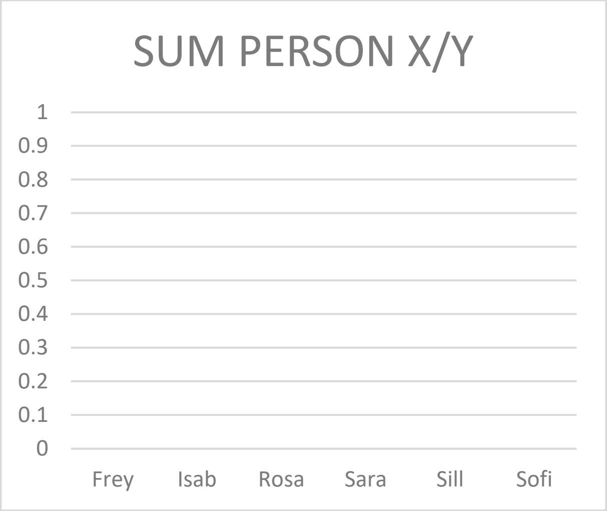

Right rot

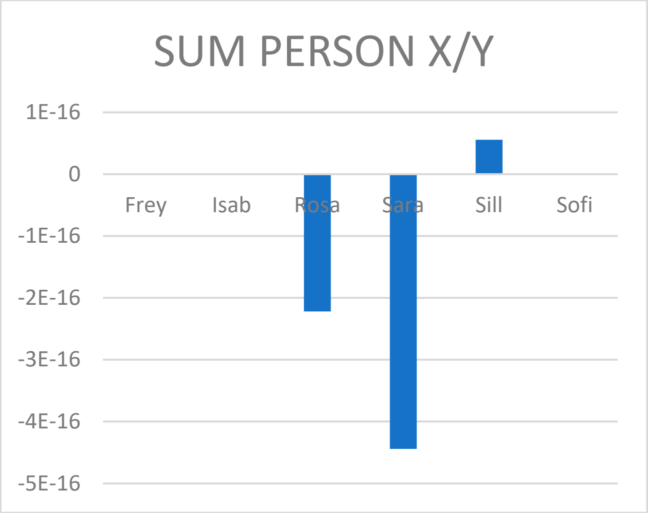

Left Lat flex

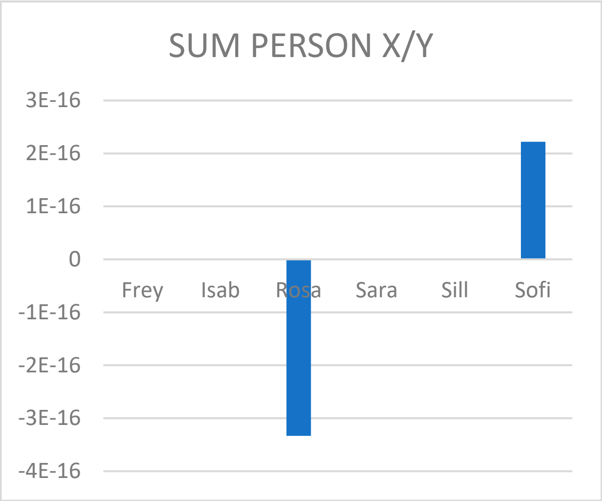

Right lat Flex

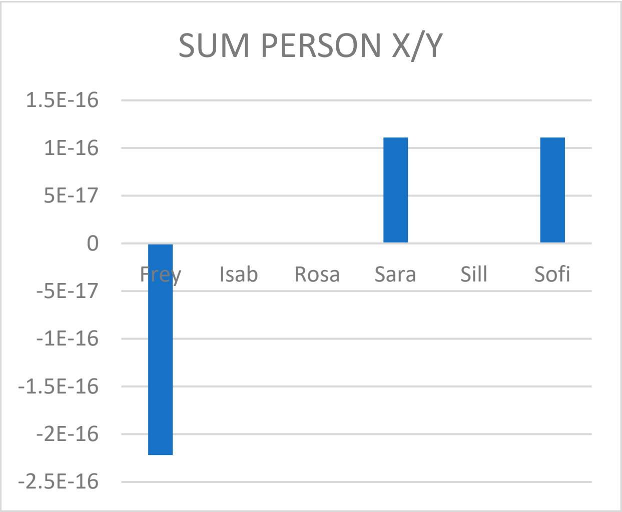

N. MEAN

Left rot

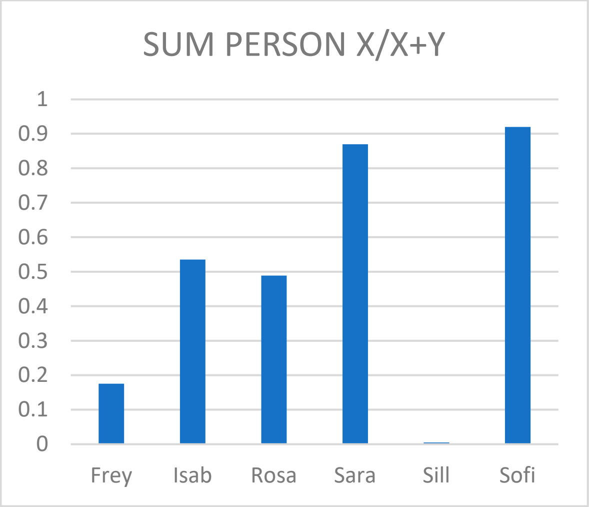

Right rot

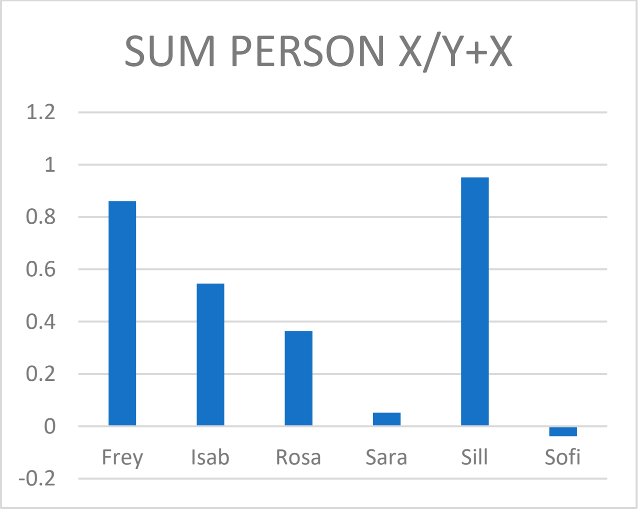

Left Lat flex

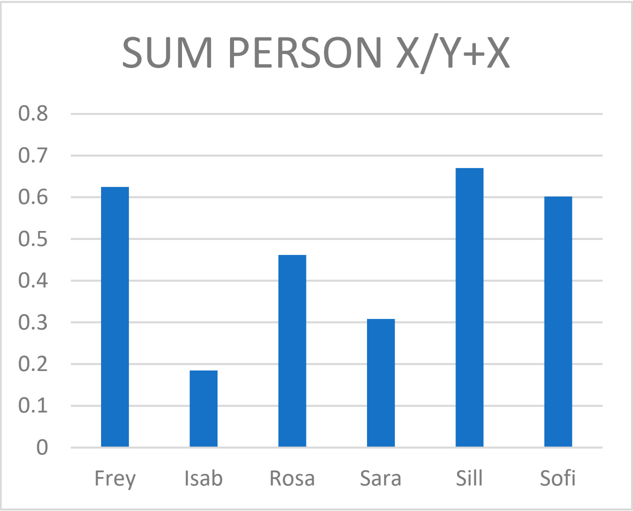

Right lat Flex

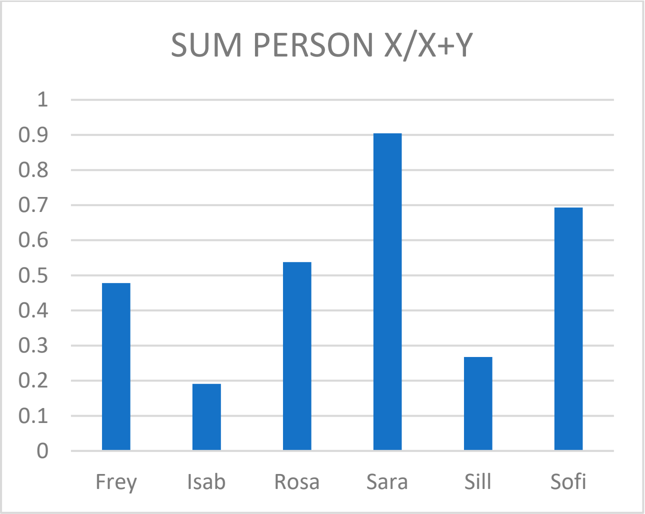

O. PEAK

Left rot

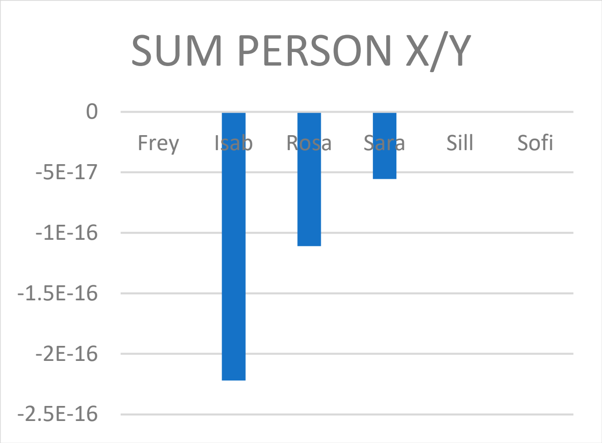

Right rot

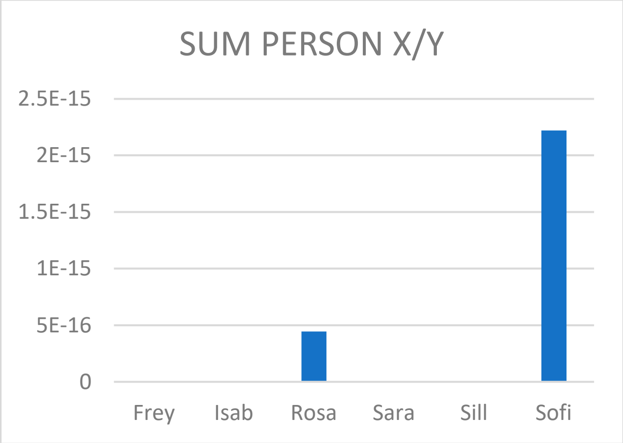

Left Lat flex

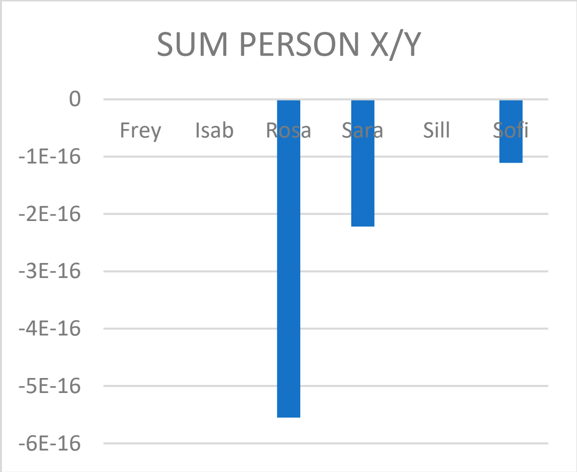

Right lat Flex

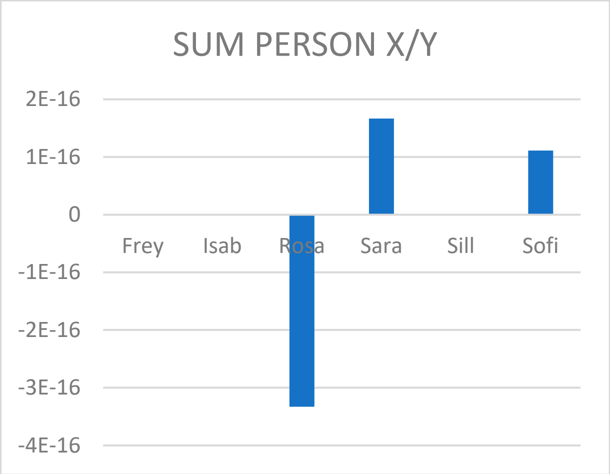

P. PEAK

Left rot

Right rot

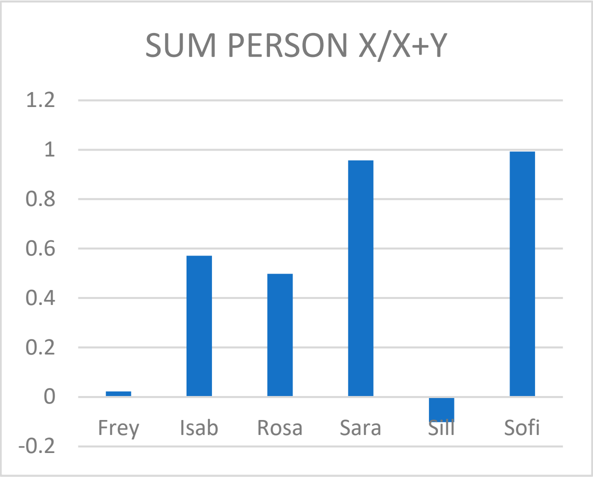

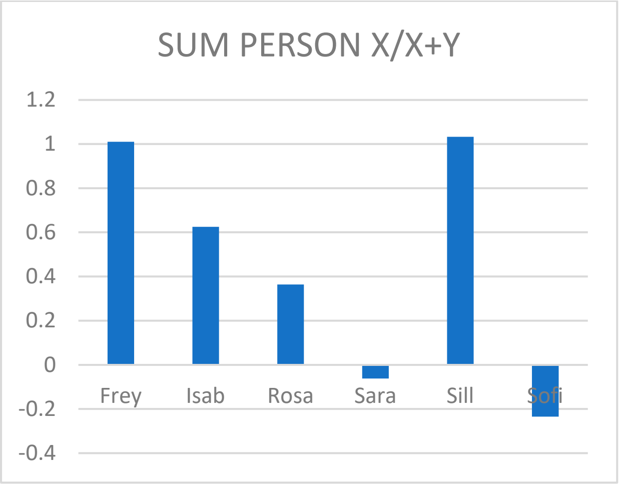

Left Lat flex

Right lat Flex

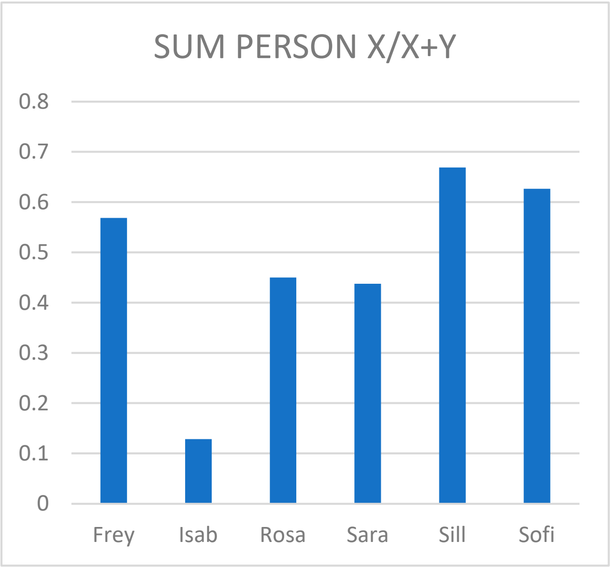

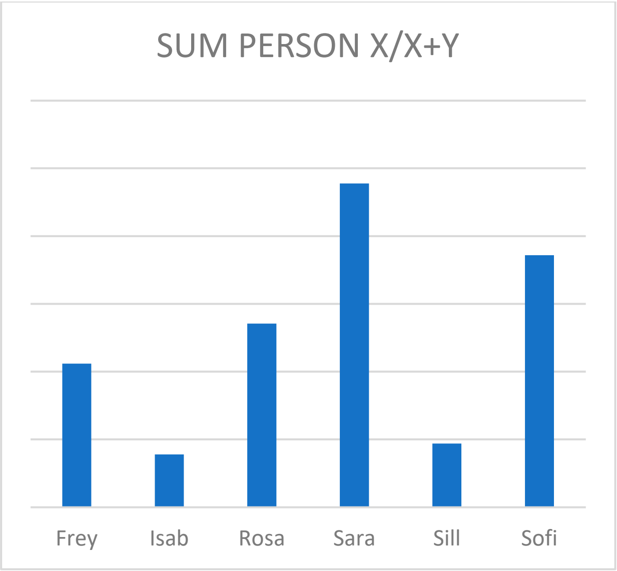

Supplement: Supplementary file 1 [file jcm-13-01758-s001.zip › Supplementary material STIM and EMG marts 24.pdf]
